# The Timing of the Circadian Clock and Sleep Differ between Napping and Non-Napping Toddlers

**DOI:** 10.1371/journal.pone.0125181

**Published:** 2015-04-27

**Authors:** Lameese D. Akacem, Charles T. Simpkin, Mary A. Carskadon, Kenneth P. Wright, Oskar G. Jenni, Peter Achermann, Monique K. LeBourgeois

**Affiliations:** 1 Sleep and Development Laboratory, Department of Integrative Physiology, University of Colorado at Boulder, Boulder, CO, United States of America; 2 College of Osteopathic Medicine, Rocky Vista University, Parker, CO, United States of America; 3 Sleep and Chronobiology Laboratory, EP Bradley Hospital, Department of Psychiatry and Human Behavior, The Warren Alpert Medical School of Brown University, Providence, RI, United States of America; 4 Centre for Sleep Research, School of Psychology, Social Work and Social Policy, University of South Australia, Adelaide, Australia; 5 Sleep and Chronobiology Laboratory, Department of Integrative Physiology, University of Colorado at Boulder, Boulder, CO, United States of America; 6 Child Development Center, University Children’s Hospital Zurich, Zurich, Switzerland; 7 Institute of Pharmacology and Toxicology, Section of Chronobiology and Sleep Research, University of Zurich, Zurich, Switzerland; Simon Fraser University, CANADA

## Abstract

The timing of the internal circadian clock shows large inter-individual variability across the lifespan. Although the sleep-wakefulness pattern of most toddlers includes an afternoon nap, the association between napping and circadian phase in early childhood remains unexplored. This study examined differences in circadian phase and sleep between napping and non-napping toddlers. Data were collected on 20 toddlers (34.2±2.0 months; 12 females; 15 nappers). Children followed their habitual napping and non-napping sleep schedules (monitored with actigraphy) for 5 days before an in-home salivary dim light melatonin onset (DLMO) assessment. On average, napping children fell asleep during their nap opportunities on 3.6±1.2 of the 5 days before the DLMO assessment. For these napping children, melatonin onset time was 38 min later (*p* = 0.044; *d* = 0.93), actigraphically-estimated bedtime was 43 min later (*p* = 0.014; *d* = 1.24), sleep onset time was 59 min later (*p* = 0.006; *d* = 1.46), and sleep onset latency was 16 min longer (*p* = 0.030; *d* = 1.03) than those not napping. Midsleep and wake time did not differ by napping status. No difference was observed in the bedtime, sleep onset, or midsleep phase relationships with DLMO; however, the wake time phase difference was 47 min smaller for napping toddlers (*p* = 0.029; *d* = 1.23). On average, nappers had 69 min shorter nighttime sleep durations (*p* = 0.006; *d* = 1.47) and spent 49 min less time in bed (*p* = 0.019; *d* = 1.16) than non-nappers. Number of days napping was correlated with melatonin onset time (*r* = 0.49; *p* = 0.014). Our findings indicate that napping influences individual variability in melatonin onset time in early childhood. The delayed bedtimes of napping toddlers likely permits light exposure later in the evening, thereby delaying the timing of the clock and sleep. Whether the early developmental trajectory of circadian phase involves an advance associated with the decline in napping is a question necessitating longitudinal data as children transition from a biphasic to monophasic sleep-wakefulness pattern.

## Introduction

Early childhood is a time of significant changes in the duration and timing of sleep [1–5]. Total 24 h sleep time declines in the early years of life, which is primarily due to a gradual reduction in napping frequency and duration [2]. Although almost all 2-year-olds meet part of their sleep need by napping, longitudinal and cross-sectional data indicate cultural differences in the age at which children consolidate sleep into one nocturnal episode. For example, about 7% of Swiss and Icelandic children are still napping at least one day per week at the age of 5 years, which differs from reports of white (60%) and black (90%) children raised in the United States [2, 6, 7]. The sleep changes observed across early childhood likely result from complex interactions of developing intrinsic bioregulatory sleep processes and extrinsic factors, including daycare and preschool schedules, parental preferences, and family demands [8–11].

The ubiquitous occurrence and gradual decline of napping in early childhood provides a rich developmental context for examining questions about sleep regulation. For example, does napping contribute to the large inter-individual variability in circadian timing observed in humans across the lifespan? How does napping influence interactions between the homeostatic and circadian processes governing sleep timing and duration? As first proposed by Borbély, the homeostatic process dictates that sleep propensity builds with increasing time awake and dissipates during periods of sleep [12]. Sleep electroencephalography (EEG) findings from adults and toddlers indicate reduced nocturnal nighttime sleep drive as a function of daytime napping (e.g., longer sleep onset latency, decreased slow wave activity in non-rapid eye movement sleep, 0.75–4.5 Hz) [13–15]. Additionally, evidence linking naps, nighttime sleep, and the homeostatic process in children is inferred from studies using parent-reports or actigraphy suggesting that preschoolers who nap longer during the day are more likely to sleep less the following night [1, 16, 17]. Although these nap-dependent results are in line with predictions made by the two-process model of sleep regulation, they speak only to the influence of napping on nighttime sleep homeostasis.

Little is known about associations between napping and the circadian timing system in early childhood. An established literature indicates that the timing of the circadian clock is influenced by environmental cues such as light and promotes alertness across the day (being highest in the early evening) [12, 18]. Circadian phase is highly variable in humans [19–23]. Even in habitually napping toddlers, we have reported a range of ~3.5 h in melatonin onset time [19]. Later circadian timing is consistently associated with delayed bedtimes, sleep onset times, midsleep times, and wake times [19, 20, 24–27]. Based upon the circadian phase dependent response to light in adults, morning light exposure shifts the circadian clock to an earlier time, whereas evening light exposure delays the timing of the circadian pacemaker [28]. In the context of known nap-dependent differences in evening sleep timing, we propose that napping indirectly influences the timing of the circadian clock via later bedtimes and the “gating” of light exposure.

In this study, we obtained a reliable marker of circadian phase, dim light melatonin onset (DLMO), and actigraphic estimates of sleep in a sample of healthy napping and non-napping 30- to 36-month-olds. Because napping has been shown to dissipate the waking homeostatic sleep drive, we expected nap status differences in a number of sleep variables. Specifically, we hypothesized that napping toddlers would have later sleep timing (i.e., bedtime, sleep onset time, midsleep time), longer sleep onset latencies (interval from bedtime to sleep onset time), and shorter nighttime sleep durations than those who did not nap. Additionally, we explored group differences in morning wake time. In terms of the circadian process, we hypothesized that napping in comparison to non-napping toddlers would have later circadian phases. Our multi-day sleep actigraphy protocol also allowed us to test the hypothesis that nap frequency (i.e., number of days napping during the 5 days before DLMO), duration, and timing would be positively correlated with melatonin onset. Finally, we explored associations between napping and phase differences (i.e., interval between DLMO and 5-day average of bedtimes, sleep onset times, midsleep times, wake times).

## Methods

Families in Providence, RI were recruited through flyers and laboratory website advertisements and at community events. All participants were healthy, normally developing toddlers with no sleep or behavioral problems. Exclusion criteria included the following: regular co-sleeping, variable sleep schedules of >2 hours between weekdays and weekends, travel across >2 time zones within 3 months of the study, regular use of medications influencing the sleep and circadian systems, diagnosed sleep problems, developmental disabilities, neurological/metabolic disorders, chronic medical conditions, lead poisoning, head injury involving loss of consciousness, conceptual age of ≤37 weeks or >42 weeks, low birth weight (<5.5 lb.) or first degree family history of narcolepsy, psychosis, or bipolar disorder. The Institutional Review Board at Brown University approved this study. Approval number: FWA00004460. All parents signed an informed consent form approved by the Brown University Institutional Review Board. Families were compensated with $50 cash, and children received a $50 United States Savings Bond upon completion of the study.

### Participants

Participants were 20 healthy children (11 females; 18 Caucasian, 1 African-American, 1 mixed-race) ages 30- to 36-months (34.2±2.0). Six toddlers attended daycare (4 full time; 2 part-time), 5 received in-home care by an extended family member or non-family member, and 9 were cared for exclusively by their parents. We initially categorized children as napping or non-napping based upon parental report of children’s habitual sleep patterns. We then verified napping status with data from actigraphy and sleep diaries during the 5 days before the DLMO assessment. Napping children (n = 15) had a biphasic sleep pattern (i.e., one daily nap opportunity and a nighttime sleep episode) and were defined as those who fell asleep during their daily nap opportunity ≥1 of the 5 days (20%) before their DLMO assessment, whereas non-napping children (n = 5) were those who slept only at night. All subjects awakened spontaneously in the morning. The napping children in this analysis represent a subset of a larger sample for which DLMO, sleep timing, and phase difference data were described [19] and were also included in a previous publication of associations between circadian parameters and nighttime settling difficulties in toddlers [29].

### Protocol

During study days 1–5, children followed their habitual sleep schedules and researchers performed several in-home training visits to ensure participants could provide adequate saliva samples for the DLMO assessment. On the sixth day, children participated in an in-home DLMO assessment performed by researchers. During the protocol, caffeine and medications affecting the sleep, circadian, and arousal systems were avoided. Researchers performed daily tracking of children’s sleep patterns and study compliance through email or telephone. Children were studied only during the fall, winter, and spring (September-May), and data were not collected within 1-week following transitions to or from daylight saving time.

### Measures

#### Sleep Diary

Parents completed a daily 26-item sleep diary throughout the study (S1 File). Diary entries were used to confirm actigraphic recordings.

#### Actigraphically-Estimated Sleep Variables

Participants wore an actigraph (model AW64; MiniMitter Company, Bend, OR, USA) on their non-dominant wrist throughout the study to provide continuous recordings of estimated sleep-wakefulness states from motor activity. Parents/caregivers pressed an event marker at the time they expected their child to try to fall asleep (lights-out) and the time their child awakened from sleep for nap and nighttime episodes. Actigraphs were downloaded on Day 6, and the actograms were inspected by researchers and reviewed with parents to verify that times from event markers were consistent with sleep diaries and/or daily call-in/email reports. This approach shows high concordance (r = 0.88, p<0.001) between sleep onset latency as measured by polysomnorgraphy and by the actigraphy in healthy preschool children [30]. Standard laboratory methods for scoring actigraphy data have been previously published [1, 19, 31, 32]. Average estimates from actigraphy were computed within individual subjects across study days 1–5 for the following variables: bedtime (lights-off time), sleep onset time, wake time (sleep end time), sleep duration (interval between sleep onset and sleep end), and sleep onset latency (interval between bedtime and sleep onset time). These mean values were used to compute the respective phase differences relative to DLMO time. Due to non-compliance, actigraphy data were not available for one child (a non-napper); in this case, we used daily diary data to compute napping, nighttime sleep, and phase difference variables.

#### Salivary Dim Light Melatonin Onset (DLMO)

Details regarding the saliva collection protocol have been previously published [19]. Briefly, children provided saliva samples (~2 mL) in dim-light (<10 lux) every 30 min for 6 h up to an hour past their average bedtime (12 samples total). Saliva was collected by having children chew on a braided dental cotton roll (Henry Schein Inc., Denver, Pennsylvania, USA) for 1–2 min. Light levels were measured and recorded for each sample using a lux meter held approximately 5 cm adjacent to the child’s eye and directed in the angle of gaze (Extech Instruments, Spring Hill, Florida, USA). Samples were immediately centrifuged (LabEssentials, Inc. Monroe, Georgia, USA), refrigerated on-site, and then frozen at the laboratory (-20°C) within 12 h. Assays were performed at the Bradley Hospital Sleep and Chronobiology Laboratory (Providence, RI, USA) using radioimmunoassay (ALPCO Diagnostics, Salem, New Hampshire, USA), minimum detection limit 0.2 pg/mL.

### Data Processing and Analysis

We used previously published methods to establish circadian phase and to compute phase differences [19, 20]. DLMO was calculated as the first interpolated time point above 4 pg/mL, with at least one subsequent sample remaining above this threshold. Phase differences were computed as the time interval between DLMO and average 5-day actigraphic estimates of bedtime, sleep onset time, midsleep time, and wake time.

Statistical analyses were performed with SPSS Statistics 21.0 (IBM Corp. Armonk, NY, USA). Summary measures are presented as *M* ± *SD*. Pearson and Spearman correlations were used to assess associations between continuous and ordinal variables, respectively. Independent t-tests were performed to investigate differences in circadian and sleep variables between napping and non-napping children. Effect size in *SD* units was computed for sleep and circadian *M* comparisons [*d* = (*M*_sample1_- *M*_sample2_)/*SD*_pooled_]. An effect size of 0.20 is considered small, 0.50 is considered medium, and ≥0.80 is considered large [33].

## Results

**Fig 1:**
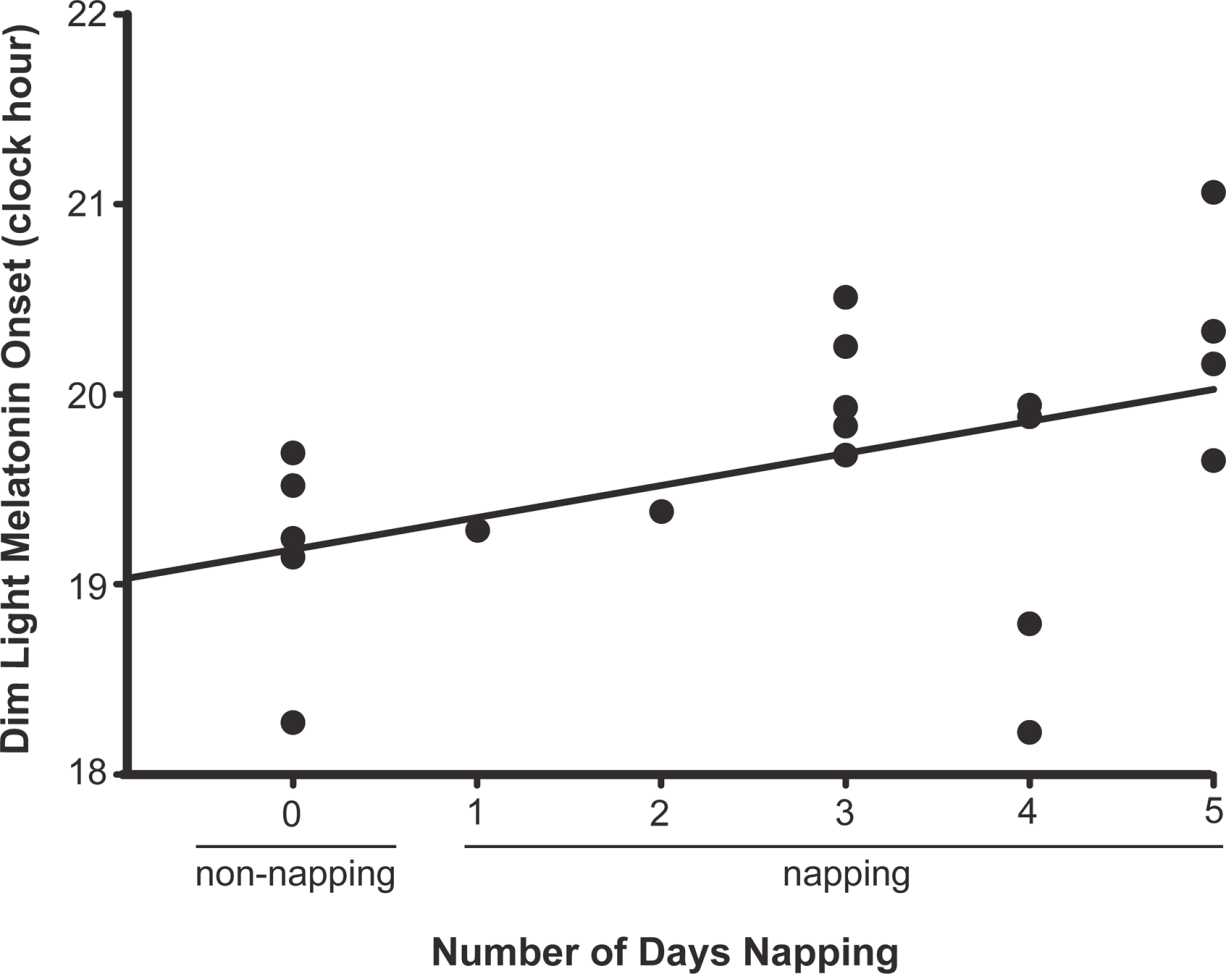
Scatterplot showing the association between napping and circadian phase in toddlers (n = 20). Number of days that toddlers napped during the 5-days preceding the dim light melatonin onset (DLMO) assessment was estimated with actigraphy (*r* = 0.49, *p* = 0.014).

Descriptive data and statistical comparisons for circadian and actigraphic sleep variables are presented in Table 1. Napping children fell asleep during their daily nap opportunities on 3.6±0.8 of the 5 days before the DLMO assessment. On average, the DLMO time of nappers was 38 min later than non-nappers (*p* = 0.044, *d* = 0.93). Napping toddlers also spent 48 min less time in bed (*p* = 0.019, *d* = 1.16) and slept an average of 69 min less at night (*p* = 0.006, *d* = 1.47); however, total 24 h sleep duration did not differ between groups (*p* = 0.156). Nappers also had 43 min later bedtimes (*p* = 0.001, *d* = 1.24) and fell asleep an average of 59 min later (sleep onset time; *p* = 0.006, *d* = 1.46) than non-nappers. On average, napping children took 16 min longer to fall asleep after bedtime (sleep onset latency) than those not napping (*p* = 0.030, *d* = 1.03). Morning wake times did not differ by napping status (*p* = 0.575). Together, this sleep pattern resulted in a non-significant trend toward earlier sleep timing in non-napping than napping toddlers, as reflected in the midpoint of sleep (*p* = 0.058). Although the bedtime, sleep onset, and midsleep phase differences did not differ based on napping status, the average wake time phase difference was narrower in napping than non-napping toddlers (*p* = 0.029, *d* = 1.23). The number of days children napped in the 5 days before the DLMO assessment was correlated with melatonin onset time, such that children who napped more often had later circadian phases (*r* = 0.49; *p* = 0.014; see Fig 1). Contrary to our hypotheses, neither nap duration nor nap midpoint time in napping subjects was associated with melatonin onset time (*r* = 0.007, *p* = 0.490; *r* = -0.36, *p* = 0.091, respectively).

**Table 1:** Descriptive statistics (Mean ± SD) for circadian and actigraphic sleep measures for napping (n = 15) and non-napping (n = 5) toddlers.

	Napping	Non-Napping	Statistics		
	(n = 15)	(n = 5)	*t*	*d*	*p*
**Circadian Variables**					
Dim Light Melatonin Onset Time	19:48 ± 0:42	19:10 ± 0:33	1.81	0.93	0.044
Bedtime Phase Difference (min)^§^	30.4 ± 33.9	25.0 ± 41.1	-0.29	0.15	0.773
Sleep Onset Phase Difference (min) ^§^	63.9 ± 32.5	42.0 ± 42.0	-1.22	0.63	0.240
Midsleep Phase Difference (min) ^§^	367.0 ± 28.6	379.6 ± 25.3	-0.87	0.45	0.394
Wake Time Phase Difference (min) ^§^	670.1 ± 39.9	717.1 ± 33.1	-2.37	1.23	0.029
**Sleep Variables**					
Bedtime	20:18 ± 0:36	19:35 ± 0:30	2.42	1.24	0.014
Sleep Onset Time	20:51 ± 0:43	19:52 ± 0:30	2.84	1.46	0.006
Midsleep Time	1:54 ± 0:30	1:30 ± 0:23	1.65	0.85	0.058
Wake Time^§^	6:58 ± 0:30	7:07 ± 0:41	-0.57	0.30	0.575
Sleep Onset Latency (min)	33.0 ± 16.6	17.1 ± 10.2	-2.01	1.03	0.030
Nighttime Time in Bed (min)	656.9 ± 38.9	705.6 ± 51.9	-2.24	1.16	0.019
Nighttime Sleep Duration (min)	606.3 ± 43.9	675.0 ± 56.2	-2.84	1.47	0.006
24-h Sleep Duration (min) ^§^	708.9 ± 40.2	675.0 ± 56.2	1.48	0.76	0.156
Days Napping (of 5)	3.6 ± 1.2	-	-	-	-
Nap Midpoint Time	14:43 ± 0:46	-	-	-	-
Nap Duration (min)	102.6 ± 20.1	-	-	-	-

Statistics are shown for independent t-tests (napping versus non-napping participants).

^§^ indicates a two-tailed test.

## Conclusions

We examined differences in sleep and circadian parameters between healthy, normally developing napping and non-napping toddlers using actigraphy as a measure of sleep and DLMO time as a marker of circadian phase. As hypothesized, we found that napping toddlers had later bedtimes, sleep onset times, and circadian timing than non-napping toddlers. Although we observed a non-significant trend towards a later midsleep time in nappers (*p* = 0.058) than non-nappers, we did not find a nap-related difference in morning wake times. Nappers also took longer to fall asleep and slept less at night as compared to their non-napping peers; however, total 24-h sleep time was similar. Although we found no nap status differences in the bedtime, sleep onset, and midsleep time relative to DLMO, nappers did show a more narrow range of wake times relative to DLMO than non-nappers. Moreover, toddlers who napped more often had later circadian phases; however, nap duration and nap timing were not associated with melatonin onset time.

In this study, evening actigraphically-estimated sleep timing was delayed in nappers compared to non-nappers, which is consistent with published observational data [34, 35]. In one study, bedtimes were reportedly later in napping than non-napping preschoolers [35], and in another, nursery school children who were required to take an afternoon nap had bedtimes that were 30 minutes later than those of the same age who did not nap [34]. These findings indicate that as children stop napping, caregivers may adjust bedtimes based upon their children’s total sleep need, readiness for sleep, and/or signs of evening sleepiness. Additionally, data from previous reports indicate that daytime naps reduce subsequent night homeostatic sleep pressure, which provides a underlying mechanism for our results of later sleep onset times, longer sleep onset latencies, and shorter nighttime sleep durations in napping than non-napping toddlers [13–15]. Our finding of no significant nap-related difference in 24 h sleep time (~30 min; *p* = 0.156; see Table 1) may suggest stability in overall sleep need as children transition from a biphasic to a monophasic sleep pattern; however, this interpretation must be taken with caution due to our small sample size.

Our findings of circadian phase differences between napping and non-napping children demonstrate that napping habits may contribute to the observed large inter-individual variability in the timing of the clock in early childhood [19]. Consistent with our hypothesis, we found that napping in comparison to non-napping toddlers had later melatonin onset times, which may in part be mediated by differences in bedtimes and sleep onset times. Delayed sleep times not only facilitate exposure to more indoor electrical lighting, but may also create a later window of time to use media and electronic devices. This is especially significant given the media-saturated context in which children are developing and recent data showing an increase in mobile electronic media use among young children [36]. Furthermore, recent experimental data indicate that the timing, duration, and intensity of light produced by electronics are important considerations in the context of understanding the timing of the circadian clock [37–40] and evening arousal levels [37, 41]. Although a detailed description of the phase shifting effects of light on circadian rhythms is not yet available for children, we would expect evening light exposure to delay the timing of the biological clock based upon established adult data [28]. It is important to note that in this study, the morning wake times of napping and non-napping toddlers were similar. Burgess and colleagues found that with a set wake time, individuals with late bedtimes had later DLMOs compared to individuals with early bedtimes, suggesting that a later bedtime facilitates a phase delay of the clock [42]. Indeed, in our recent work on napping toddlers, we showed that sleep timing and DLMO were positively correlated, such that later bedtimes, sleep onset times, midsleep times, and wake times were associated with a later timing of evening melatonin onset [19].

Although the adolescent circadian phase delay is well established, earlier developmental changes in the timing of the clock are unknown [43, 44]. In our previously published work, we found an average DLMO time of 19:29 in a sample of 45 napping toddlers, whereas a DLMO of 20:42 was reported in 9 year-old children who were assessed during the academic year [19, 23]. This age-related phase delay continues later into the second decade of life, with mature adolescents exhibiting an average DLMO of 21:53 [23]. Although the current literature points to a delay in circadian phase between early and late childhood, findings from this study suggest that circadian phase may first advance as children begin to drop their naps. We not only found that non-nappers had ~35 min earlier melatonin onset times than nappers, but also that napping less frequently was associated with earlier DLMOs. In sum, these results suggest a phase advance in the timing of the circadian clock may occur as children give up their naps; however, longitudinal data of circadian phase during the transition from a biphasic to monophasic sleep pattern are needed to address this important ontogenetic research question.

The cessation of napping typically unfolds gradually during early childhood. Observational findings indicate that naps become less frequent (days per week), shorter, and occur at later times of the day with increasing age [2, 10, 45, 46]. As hypothesized, we found that napping frequency was positively correlated with melatonin onset time. Napping more often across a week may have cumulative effects on melatonin onset via associated later bedtimes, which gate evening light exposure. Because experimental data in adults indicate that morning naps delay circadian phase, evening naps advance the clock, and afternoon naps have no influence on melatonin onset time [47], we hypothesized that nap timing would be associated with circadian phase. We also expected children taking longer naps to have later melatonin onset times due to greater dissipation of sleep propensity during the daytime nap episode [13–15]. Neither relationship was supported by our findings, which may have been due to little variability in nap timing and nap duration. That is, in our sample of 30- to 36-month-olds, naps occurred in the afternoon, almost 12 hours after nocturnal midpoint of sleep, a time at which we would expect the clock to be less sensitive to phase shifts. Similarly, nap duration was on average about 100 minutes, with a narrow range (~83 minutes). Assessing the influence of multiple nap dimensions on the circadian clock will necessitate longitudinal data that provide more sensitive trajectories of shifts in napping patterns across early childhood.

Phase differences provide an approach to quantifying associations of the sleep and circadian systems. In this study, we did not observe nap status differences in the bedtime, sleep onset, or midsleep phase differences. Napping children, however, had longer sleep onset latencies than those who had given up their naps. Together, this pattern of findings highlights the interaction between the homeostatic and circadian processes in determining sleep propensity and sleep timing. In regularly napping toddlers, more narrow bedtime phase differences are associated with longer sleep onset latencies [29], indicating the importance of the circadian timing of sleep when evening levels of homeostatic sleep pressure are more-or-less similar. Our current analysis comparing napping and non-napping children showed that children were being put to bed at the same circadian time, regardless of their napping status. Thus, although the interval between melatonin onset and bedtime is important in determining sleep onset latency in napping children, it may make a smaller contribution once children give up their naps. At this developmental transition, the build up of homeostatic sleep pressure may play a stronger role in promoting levels of evening sleepiness than the circadian timing of sleep. Additionally, we found napping status differences in the wake time phase difference, which can be explained by the earlier DLMO times observed and longer nocturnal sleep in the non-nappers and the similar wake times between napping and non-napping children. Although our methods did not permit measurement of the length of the biological night, our data suggest that napping children may wake up at an earlier circadian time compared to their non-napping peers. Whether this nap-related pattern influences variability in sleep inertia, alertness, cognitive performance, or mood in the morning hours is an important future research direction with social and academic implications.

Shifting from a napping to a non-napping sleep schedule is not always a smooth developmental transition. Many toddlers and preschoolers naturally drop their naps; however, about one-third are forced to stop napping due to parental preferences and preschool or daycare schedules [10]. The compulsory nighttime consolidation of sleep may have advantages and disadvantages for parents and children. For example, recent data indicate that the transition to full-day kindergarten influences the sleep patterns of children, including a sharp decline in weekday napping, an advance in nocturnal sleep timing, and parent-reports of fewer difficulties going to bed and falling asleep [48]. Moreover, our finding of longer sleep onset latencies in napping children suggests that removing daytime naps may be beneficial to children who exhibit evening settling difficulties (e.g., prolonged sleep onset, bedtime resistance), which are reportedly reasons for compulsory consolidation by ~30% of U.S. parents [10]. On the other hand, certain children who are required to stop napping may not be able to adapt to associated increases in homeostatic load and may suffer from daytime sleepiness, poor daytime functioning, or parasomnias such as night terrors [31, 49–52]. For instance, one study reported a nap-dependent improvement on recall tests in habitual but not non-habitual napping preschoolers [53]. Furthermore, our previously published results indicate that taking away one nap of ~90 minutes impairs toddlers’ emotion processing and self-regulation strategies [31, 51]. Based upon these nap-dependent functional data, individual differences in the biologically optimal time of nighttime sleep consolidation likely exist. The development of different strategies for phasing out naps that maintain their cognitive and emotional benefits as well as reduce associated evening settling difficulties represents an important area of future research.

We interpret our findings of napping-associated differences in evening sleep timing, sleep onset latency, and circadian phase in the social context in which children develop. For example, findings from a number of studies indicate that the age at which the majority of children stop napping varies across cultures. In Iceland, the proportion of children who nap decreases early in life, with ~90% napping at age 2 years and only ~22% napping at age 3 years [7]. In Japan and Switzerland, the proportion of children who nap is ~85% at 2 years and ~50% by the age 3 years [2, 35]. In contrast, napping cessation occurs on average at a later age in children raised in the United States and varies across ethnicities. In one Chicago-based longitudinal study, ~70% of 3-year-olds and ~30% of 4-year-olds were reported by their parents to nap [10]. Furthermore, another study of children living in Southern Mississippi found that the majority of white children were no longer napping between ages 5 and 6 years, while the transition to exclusive nighttime sleep in black children occurred about one year later [6]. Institutional demands such as those put in place by early childcare settings can also influence the napping habits of young children [11]. Daycares and preschools may or may not enforce a “quiet time” or nap period, and the duration of nap opportunities may be developmentally inappropriate or occur at a time too late in the day with respect to the bedtimes parents select for their children. A recent emphasis on early academic performance and school readiness has also resulted in a number of early childhood centers replacing naptime with learning activities [8, 54]. As more than 70% of American children between the ages 2 and 5 years attend center-based care, institutional napping policies impact a significant proportion of U.S. children [55]. Thus, the context in which children develop (e.g., culture, daycare, preschool, home care) plays an important social role in the development of sleep patterns, the emergence of nighttime settling difficulties, and observed inter-individual variability in the timing of evening sleep patterns and melatonin onset times.

Although this study represents an important step in understanding sleep behavior and circadian physiology in early childhood, it has several limitations. Our sample size was small with 15 napping children and 5 non-napping children. In general, this ratio reflects the proportion of napping and non-napping toddlers; however, a larger sample size would strengthen our conclusions. Furthermore, we enrolled only healthy, good-sleeping toddlers. Considering the prevalence of behavioral sleep problems in childhood, our findings may not be reflective of the general population. Finally, our analytic approach permitted comparisons solely between nappers and non-nappers, which limits the interpretation of more time-sensitive, developmental trajectories of sleep patterns and the timing of the circadian system as a function of multiple nap dimensions (i.e., duration, timing, frequency).

In summary, our results suggest that napping in toddlerhood is not only associated with later evening sleep timing, shorter nighttime sleep duration, and longer sleep onset latencies, but also later timing of the internal circadian clock. Given the increase in media exposure in childhood, as well as the importance of light in determining melatonin phase, future experimental and naturalistic studies examining the influence of light exposure on the circadian system of toddlers and preschoolers are warranted. Time-sensitive, longitudinal research assessing concurrent changes in the sleep and circadian systems and daytime functioning during the transition from a biphasic to monophasic sleeping pattern is also needed to understand the fundamental question of the optimal timing of nighttime sleep consolidation in early childhood.

## Supporting Information

S1 Dataset Subject demographics and sleep and circadian timing data. Excel file with data used in the present analysis. Columns are: ID, sex, age (months), DLMO time (decimalized time), days napped (out of 5), napping status (0 = non-napper; 1 = napper), nap lights out time (decimalized time), nap sleep onset time (decimalized time), nap midsleep time (decimalized time), nap sleep offset time (decimalized time), nap wake time (decimalized time), nap duration (minutes), nap time in bed (minutes), night bedtime (decimalized time), night sleep onset (decimalized time), sleep onset latency (hours), night midsleep time (decimalized time), night wake time(decimalized time), night sleep duration (minutes), night time in bed (minutes), 24 h sleep duration (minutes), bedtime phase difference (hours; negative value indicate subject was put to bed after DLMO), sleep onset phase difference (hours; negative value indicate subject was put to bed after DLMO), midsleep phase difference (hours), wake time phase difference (hours).
(XLSX)

S1 File Sleep diary. 26-item sleep diary completed daily by parents throughout the study.
(DOCX)
