# Supplementary material for: The Timing of the Circadian Clock and Sleep Differ between Napping and Non-Napping Toddlers
Source: PLoS One. 2015 Apr 27;10(4):e0125181. doi: 10.1371/journal.pone.0125181 (PMC4411103; doi:10.1371/journal.pone.0125181)
Supplement: S1 File — 26-item sleep diary completed daily by parents throughout the study. (DOCX) [file pone.0125181.s002.docx]

| Day of Week (circle): Mon Tues Wed Thurs Fri Sat Sun ID: _____________ Date:__________________ |
| --- |
| **ANSWER 1 – 9 JUST AFTER YOUR CHILD WAKES UP**   1. *Number of times child awakened during the night*: ___ 0 ___ 1 ___ 2 ___ 3 ___ 4+ *For a total of*  ______ minutes 2. *Child’s sleep disturbed last night by* (*you can check more than one*): ___ Did not wake during night ___ Noise ___ Bathroom ___ Body aches/sick   ___ Too hot or cold ___ Other person ___ Other _______________________   1. *Did child get enough sleep last night*? ___ No way! Not enough sleep ___ Sort of the right amount ___ Yes! S/he slept the needed amount 2. *Overall, child’s sleep last night was*: ___ Very poor ___ Poor ___ Okay ___ Good ___ Very good 3. *Did child awaken before you today*? ___ Yes ___ No 4. *Time child finally woke up today: ________________* AM / PM (circle one) 5. *Child was awakened by*: ___ Just woke up ___ Noise ___ Parent/other person ___ Alarm ___ Other _________________________________ 6. *Time child finally got out of bed today*: _______________ AM / PM (circle one) 7. *This morning child felt*: ___ Very tired ___ A little tired ___ Somewhat awake and rested ___ Wide awake and well-rested   **STOP! Finish The Rest Of The Questions Later** |
| **ANSWER 10-26 AFTER CHILD FALLS ASLEEP AT NIGHT**   1. *Did any of these happen today*? ___ None happened ___ Holiday/vacation ___ Child attended school/daycare ___ School/day care delayed   ___School/daycare cancelled ___ Child sick ___ Other event ________________________________________________________________________   1. ***Overall****, child’s mood today: ____* Very bad mood ____ Sort of Bad mood ____ Pretty good mood ____Very good mood 2. ***Overall****, child’s level of alertness today:* ___ Very tired ___ A little tired ___ Somewhat alert/energetic ___ Very alert/energetic 3. *Any stress today for child*? ___ Almost none ___ A little stress ___ A medium amount of stress ___ A lot of stress 4. *Any excitement today for child*? ___ Almost none ___ A little excitement ___ A medium amount of excitement ___ A lot of excitement 5. *Any* ***scheduled*** *nap opportunities during the day?* ___ Yes ___ No nap opportunity   Start time? _______________ AM / PM End Time? _______________ AM / PM (circle one) Where? ________________________  Did your child fall asleep? ___Yes ___No   1. *Any accidental naps/sleep during the day?* ___ No accidental nap today OR   ___Start time? _______________ AM / PM End Time? _______________ AM / PM (circle one) Where? ________________________   1. *Child’s mood after waking from nap today:* ___ Very bad mood ___ Sort of a bad mood ___ Pretty good mood ___ Very good mood ___N/A 2. *Any caffeine today*? ___ No caffeine today OR   ___ Caffeine #1: Time? _______________ AM / PM Type? _________________ How much? _______________  ___ Caffeine #2: Time? _______________ AM / PM Type? _________________ How much? _______________   1. *Any medicine today*? ___No medicine today OR   ___Medicine #1 Time? ______________ AM / PM Type? ___________________________________________  ___ Medicine #2: Time? ______________ AM / PM Type? ___________________________________________   1. *Took watch off today because:* ___ Did not take the watch off today OR   Time Off? _______________ AM / PM Time Back On? ____________ AM / PM Reason: _______________________  Time Off? _______________ AM / PM Time Back On? ____________ AM / PM Reason: _______________________  Time Off? _______________ AM / PM Time Back On? ____________ AM / PM Reason: _______________________   1. *Tonight, child got into bed at (time):* _________________________ AM / PM (circle one) 2. *Lights out/child tried to fall asleep at (time):* ________________________ AM / PM (circle one) 3. *Length of time (minutes) for child to fall asleep after turning the lights out*: ____­­__ minutes 4. *In the last hour before “lights out” child did the following (check all that apply)*  \| ___ Played on computer / iPad  ___ Played video games  ___ Watched TV  ___ Played with games / toys  ___ Listened to music  ___ Ate dinner  ___ Ate snack  ___ Shower/bath \| ___ Brushed teeth  ___ Washed face  ___ Quiet activity like reading  ___ Played with brother/sister/parent/other person  ___ Felt strong positive emotions (excited, happy)  ___ Felt strong negative emotions (sadness, anger)  ___Other_________________________________ \| \| --- \| --- \|  1. *The LAST activity child did before “lights out” (trying to sleep) was*: ______________________________________________ How long? _______ mins |
| 1. Tell us something “special” that happened today (e.g., went to a movie, visited a friend, went out to eat, etc.):   _____________________________________________________________________________________________________  **STOP!!! End Of Diary For Today** |
